# Synthesis and Cytotoxic Activity of a New Group of Heterocyclic Analogues of the Combretastatins

**DOI:** 10.3390/molecules19067881

**Published:** 2014-06-11

**Authors:** Alla V. Lipeeva, Elvira E. Shults, Makhmut M. Shakirov, Mikhail A. Pokrovsky, Andrey G. Pokrovsky

**Affiliations:** 1 Laboratory of Medicinal Chemistry, Novosibirsk Institute of Organic Chemistry, Siberian Branch, Russian Academy of Sciences, Lavrentjev Avenue 9, Novosibirsk 630090, Russia; E-Mails: mond_05@list.ru (A.V.L.); mmsh@nioch.nsc.ru (M.M.S.); 2 Medicinal Department, Novosibirsk State University, Pirogova St. 2, Novosibirsk 630090, Russia; E-Mails: miha.pokrovsky@gmail.com (M.A.P.); decan@medf.nsu.ru (A.G.P.)

**Keywords:** furocoumarins, oreoselone, psoralen, Sonogashira coupling, semi-hydrogenation, combretastatins, cytotoxicity

## Abstract

A series of new analogs of combretastatin A-4 (CA-4, **1**) with the A or B-ring replaced by a 3-oxo-2,3-dihydrofurocoumarin or a furocoumarin residue have been designed and synthesized by employing a cross-coupling approach. All the compounds were evaluated for their cytotoxic activity with respect to model cancer cell lines (CEM-13, MT-4, U-937) using conventional MTT assays. Structure-activity relationship analysis reveals that compounds **2**, **3**, **6**–**8** in which the (*Z*)-styryl substituent was connected to the 2-position of the 3-oxo-2,3-dihydrofurocoumarin core, demonstrated increased potency compared to 3-(*Z*)-styrylfurocoumarins **4**, **5**, **9**–**11**. The methoxy-, hydroxyl- and formyl- substitution on the aromatic ring of the (*Z*)-styryl moiety seems to play an important role in this class of compounds. Compounds **2** and **3** showed the best potency against the CEM-13 cell lines, with CTD_50_ values ranging from 4.9 to 5.1 μM. In comparison with CA-4, all synthesized compounds presented moderate cytotoxic activity to the T-cellular human leucosis cells MT-4 and lymphoblastoid leukemia cells CEM-13, but most of them were active in the human monocyte cell lines U-937.

## 1. Introduction

**Figure 1:**
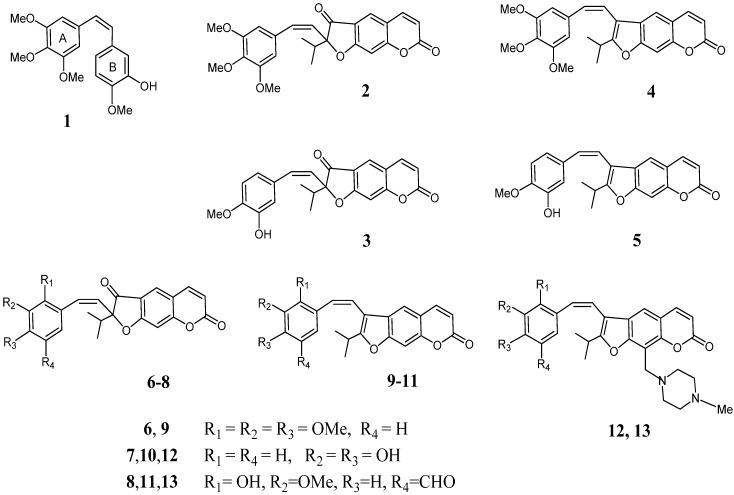
Structures of combretastatin A-4 (**1**) and furocoumarin analogs of combretastatins **2**–**13**.

Combretastatin A-4 (CA-4, **1**, Figure 1), isolated from the bark of South African tree *Combretum caffrum* [1] is one of the well-known natural tubulin-binding molecules affecting microtubule dynamics by binding to the colchicine site [2]. CA-4 (**1**) shows strong cytotoxic activity against a wide variety of human cancer cell lines, including those that are multidrug resistant [2]. A water soluble disodium phosphate derivative of CA-4 (CA-4P, fosbretabulin) has shown promising results in human cancer clinical trials [3,4], thus stimulating significant interest in a variety of CA-4 analogues [5]. 

Different analogs of combretastatins are obtained, including heterocombretastatins containing heterocyclic fragments as rings A or B [6,7,8,9,10,11,12]. A considerable cytotoxicity and antitubulin activity was revealed for heterocombretastatins containing a (4-methoxypyridin-3-yl)- or a (1-methyl-2-oxo-1,2-dihydropyridin-4-yl) ring B [6]. Compounds with benzo[*b*]furan ring B [9,10] or A [10], attached to the 6 position, demonstrated high antitumor activity in *in vitro* and *in vivo* models. In more recent works, replacement of the B-ring of CA-4 (**1)** with a (benzofuran-2-yl) moiety was also examined and the activity of the benzofuranyl derivatives was evaluated [11,12].

Herein we describe the first synthesis of combretastatin A-4 analogues **2**,**3** and **4**,**5** by replacement of the A or B aromatic ring with linear dihydrofurocoumarins or furocoumarins (psoralens) (Figure 1). The (*Z*)-styryl moiety was attached to 2- or 3-position of the heterocyclic molecule. We also include other examples of (*Z*)-arylvinylfurocoumarins **6**–**11** for obtaining structure-cytotoxicity relationships.

Linear furocoumarins are employed in Psoralen + UVA (PUVA) therapy for the treatment of autoimmune or hyper-proliferative skin diseases, including psoriasis and vitiligo. Activated by UV-A light furocoumarins induce many biological effects, such as photocycloadditions to DNA, immune system modulation, reactions with proteins, RNA and lipids [13,14,15]. In last decades many new potential therapeutic applications for furocoumarins are found. For instance, some psoralen derivatives were found to induce erythroid differentiation in different cellular models [16,17]. Moreover it was shown that annelation of a cyclopentane, cyclohexane, benzene, or pyridazine ring to the furan ring in the psoralen skeleton changed the phototoxicity and, in some cases led to a marked increase in the photo-antiproliferative activity [18]. There was therefore value in a targeted preparation and investigation of novel furocoumarins, containing a (*Z*)-styryl substituent in the furan ring of the molecules. Compounds **12** and **13**, containing an additional N-methylpiperazinyl substituent in the furocoumarin scaffold were also prepared and investigated, since modification in the 9-position has a great influence on the properties of psoralenes as enzymatic inhibition and human keratinocyte proliferation [13].

## 2. Results and Discussion

### 2.1. Chemical Synthesis

**Scheme 1:**
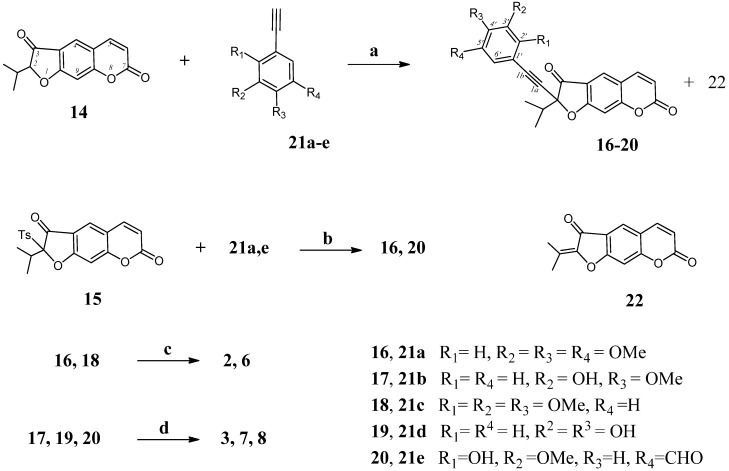
Synthesis of 2-(*Z*)-styrylfurocoumarins **2**, **3**, **6**, **7**, **8**.

The synthetic route followed for the synthesis of the desired novel combretastatin A-4 analogs **2**–**3**, and **6**–**8** is outlined in Scheme 1. Previously we found that the plant coumarin oreoselone (**14**) by reaction with *p*-toluenesulfonyl chloride gave 2-(tosyl)oreoselone (**15**), which showed a high activity in Pd-catalyzed desulfonative cross-coupling reactions [19]. Herein, oreoselone (**14**) was converted into 2-(arylethynyl)furocoumarins **16**–**20** by the copper-free cross-coupling reaction with arylalkynes **21a**-**e** in the presence of *p*-toluenesulfonyl chloride. After the purification by column chromatography compounds **16**–**20** were isolated in 44%–62% yield. 2-(Propan-2-ylidene)-7*H*-furo[3,2-*g*]chromene-3,7(2*H*)-dione (**22**) [20] was also isolated with 10%–25% yield, presumably after elimination of *p*-toluenesulfinic acid from the *in situ* formed 2-(tosyl)oreoselone (**15**). Alkynes **16** and **20** were also prepared by the cross-coupling reaction of previously synthesized 2-(tosyl)oreoselone (**15**) with arylalkynes **21a**,**e** in a THF solution in the presence of a base and a catalytic amounts of *trans*-dichlorobis(triphenylphosphine)palladium(II) (yield 59%–66%). Additionally, compound **22** was also isolated in the yield 5%–7%. To obtain heterocombretastatins containing a furocoumarin residue, we have studied the reduction of 2-(arylethynyl)furocoumarins **16**–**20**. Several protocols (alkyne hydroboration [21], hydrolysis of a preformed Ti(II)-diarylalkyne complexes [22], or Lindlar’s semi-hydrogenation [23]) are employed for the selective transformation of diarylalkynes into *cis* alkenes in the synthesis of CA-4 **1**. Treatment of alkynes **16**, **18** with Ti(O-*i*Pr)_4_ (2 equiv) and *n*-BuLi (4 equiv.) at −78 °С in THF and then increasing the temperature to 50 °С and keeping the reaction mixture at this temperature for 2 h (for hydrolysis of the formed complexes) afforded 2-(*Z*)-(styryl)furocoumarins **2**, **6** (yield 40%–42%). 2-(*Z*)-(Styryl)furocoumarins **3**, **7** and **8** were obtained by partial hydrogenation of alkynes **17**, **19** and **20** (isolated in 32%, 24% and 37% yield, respectively, after column chromatography) using Lindlar’s catalyst (Scheme 1).

*Reagents and Reaction Conditions*: (**a**): oreoselone (**14**), TsCl, Pd(PPh_3_)_2_Cl_2_ (5 mol %), THF, reflux, 6 h, then alkyne **21**, Et_3_N, THF, reflux, 7–8 h; (**b**): 2-tosyloreoselone (**15**), alkynes **21a**,**e**, Pd(PPh_3_)_2_Cl_2_ (10 mol %), Et_3_N, THF, reflux, 8 h; (**c**) Ti(O-*i*Pr)_4_–n-BuLi, THF, −78 °С, then 50 °С, 2 h; (**d**): H_2_, 1 atm, Lindlar’s catalyst, EtOH, rt, 20–30 h.

**Scheme 2:**
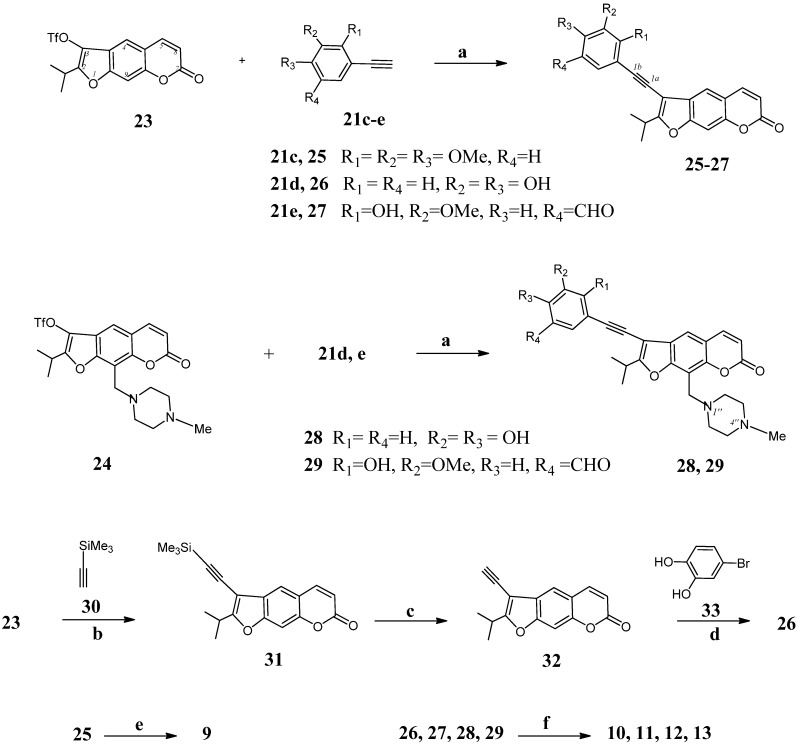
Synthesis of 3-(*Z*)-styrylfurocoumarins **9**–**13**.

3-(Z)-(Styryl)furocoumarins **9**, **10**, **11**, **12** and **13** (Scheme 2) are obtained from 2-isopropyl-3-(trifluoromethanesulfonyloxy)psoralene (**23**) [24] or 2-isopropyl-9-(4-methylpiperazin-1-ylmethyl)-3-(trifluoromethanesulfonyloxy)psoralene (**24**) [25], by approaches described early for preparation of compounds **4**, **5** [26]. The reaction of triflates **23** with arylalkynes **21c**–**e** in a benzene (*WARNING—this solvent is a known carcinogenic substance*) solution in the presence of a catalytic amounts of *trans*-dichlorobis(triphenylphosphine)palladium(II), copper(I) iodide, and triethylamine led to the corresponding 3-(arylalkyne)furocoumarins **25**–**27** in the yield 68%–73% after the column chromatography. By using the mentioned cross-coupling conditions for reacting of psoralen derivatives **24** with the alkynes **21d**,**e**, compounds **28**, **29** are obtained (yield 42%–58%). For preparation of compound **26** we employed another approach. At first, by the Sonogashira coupling of oreoselone triflate **23** with (trimethylsilyl)acetylene **30** we obtained 3-(trimethylsilyl)-2-isopropyl-psoralene (**31**) (yield 58%). Desilylation of compound **31** produced the corresponding 3-ethynyl-2-isopropylpsoralen (**32**) (66% yield). Hydrolysis of *in situ* formed Ti(II)-alkyne complex of alkyne **25** gave the 3-(*Z*)-(2,3,4-trimethoxystyryl)furocoumarin (**9**) (yield 60%). Partial reduction of alkynes **26**–**29** using Lindlar’s catalyst resulted in the isolation of 3-(*Z*)-styrylsubstituted furocoumarins **10**, **11**, **12**, or **13** (yield 40%–60%) (Scheme 2).

*Reagents and Reaction Conditions*: (**a**): triflate **23**, or **24**, Pd(PPh_3_)_2_Cl_2_ (4 mol %), CuI (2 mol %), Et_3_N, alkynes **21c**–**e**, PhH, reflux, 8–10 h; (**b**): triflate **23**, alkyne **30**, Pd(PPh_3_)_2_Cl_2_ (4 mol %), CuI (2 mol %), Et_3_N, PhH, reflux, 10 h; (**c**): CsF, MeOH, benzyltrimethylammonium chloride, rt; (**d**): Pd(PPh_3_)_2_Cl_2_ (4 mol %), CuI (2 mol %), Et_3_N, bromide **33**, PhH, reflux, 10 h; (**e**): Ti(O-*i*Pr)_4_–*n*-BuLi, THF, −78 °С, then 50 °С, 2 h; (**f**): H_2_, 1 atm, Lindlar’s catalyst, EtOH, rt, 30 h.

The structure of the synthesized compounds was established based on the combination of IR, UV, and NMR spectral data. The IR spectra of compounds **16**–**20**, **25**, **26**, **27**, **28**, **29**, **31** and **32** is characterized by the presence of the absorption band of the alkyne linker group at 2046–2191 cm^−1^. The ^1^H and ^13^C-NMR spectra of all synthesized compounds agree well with their structure and contain one set of characteristic signals of psoralen (3-oxo-2,3-dihydrofurocoumarin) skeleton and the corresponding substituent. The spin-spin coupling constants between alkenyl proton signals H-1a and H-1b (*J* 8.8–9.5 Hz) indicate the formation of compounds **2**–**13** with the (*Z*)-configuration of the double bond.

### 2.2. Cytotoxicity Studies

The cytotoxic activity of the synthesized series of 2-(*Z*)-styryldihydrofurocoumarins **2**,**3**,**6**–**8**, 3-(*Z*)-styrylfurocoumarins **4**,**5**,**9**–**13**, the parent oreoselone (**14**) and combretastatin A-4 (**1**) was determined by measuring the concentration inhibiting human tumor cell viability by 50% (CTD_50_). The CTD_50_ was determined using the conventional MTT assay, which allows to estimate the number of survived cells spectrophotometrically [27]. The results are presented in Table 1. At first glance, it is evident that there is a difference in activity between the parent compound **14** and (*Z*)-styryl modified furocoumarins **2**–**13**. 

**Table 1:** Cytotoxic activity of heterocyclic analogs of combretastatins **2**–**13**.

Compound	Cytotoxicity (CTD_50_, μM) against Cell Line *^a^*		
	CEM-13	MT-4	U-937
2	4.9 ± 0.2	13.4 ± 4.5	12.1 ± 2.0
3	5.1 ± 0.9	10.1 ± 2.1	18.0 ± 2.9
4	28.6 ± 2.1	22.4 ± 2.6	41.3 ± 6.8
5	29.0 ± 3.2	36.1 ± 4.6	32.1 ± 3.5
6	22.1 ± 2.3	28.6 ± 3.7	33.1 ± 3.9
7	9.3 ± 2.6	9.2 ± 0.5	12.0 ± 0.8
8	14.1 ± 1.6	12.1 ± 3.2	13.3 ± 2.1
9	95.0 ± 3.4	24.5 ± 3.2	>100
10	49.1 ± 4.5	13 ± 2.8	29.0 ± 3.1
11	26.7 ± 2.9	45.1 ± 1.9	28.0 ± 4.2
12	20.2 ± 1.6	5.2 ± 0.8	9.1 ± 1.2
13	9.0 ± 2.6	8.1 ± 0.7	12.1 ± 2.3
(14) CA-4 (1)	>100 0.8 ± 0.03	70.9 ± 3.6 0.1 ± 0.012	65.2 ± 3.9 >100 *^b^*

*^a^* The cell were continuously treated with compounds for 72 h; *^b^* СА-4 provide inhibition of 47% in concentration 0.1–100 μM.

All synthesized compounds **2**–**13** exhibited cytotoxic activity in respect to model cancer cell lines and were more potent than furocoumarin **14**. Furocoumarins **4**, **5**, **9**, **10** and **11**, in which the (*Z*)-styryl substituent was located in the 3-position, demonstrated decrease of potency compared to 2-(*Z*)-styryl-3-oxodihydrofurocoumarins **2**, **3**, **6**, **7** and **8**. Compounds **2**, **3** possessed the best activity on the lymphoblastoid leukemia cells CEM-13. The phenolic substituent in (*Z*)-styryl moiety seems to have an important role in this class of compounds; indeed 3,4-dihydroxyphenyl substituent in compounds **7**, **10**, **12** demonstrated increase of potency against MT-4 cell lines. Compounds having а 2,3,4-trimethoxyphenyl moiety (compounds **6**, **9**) show week cytotoxic activity, while several derivatives containing a 3,4,5-trimethoxyphenyl residue (compounds **2**, **4**) shown improved activity. The latter effect is unsurprising, since it is well established that the replacement of A-ring in combretastatins [28,29] and phenstatin [30] is highly detrimental for the activity of the compounds. In the 3,9-disubstituted furocoumarins **12**,**13** cytotoxicity does not seem to greatly influence by the substituent in the aromatic ring, however, the (4-methylpiperazin-1-ylmethyl) substitution on the 9th-position in 3-styrylfurocoumarin core gave increase in cytotoxic activity of compounds (compare **10** and **12** or **11** and **13**), especially, on the human monocyte-lines U-937. The activity of all synthesized compounds on the lymphoblastoid cell lines was lower than the activity of СA-4 **1**, however, compounds **2**,**7**,**12** and **13** shown potency in respect to human monocyte-lines U-937 in comparison with СA-4.

## 3. Experimental Section

### 3.1. General

NMR spectra were acquired on Bruker AV-400 (^1^H: 400.13 MHz, ^13^C: 100.78 MHz) or Bruker AV-600 (^1^H: 600.30 MHz, ^13^C: 150.95 MHz) (Bruker BioSpin GmbH, Rheinstetten, Germany) instruments, using tetramethylsilane (TMS) as an internal standart. In the description of the ^1^H and ^13^C-NMR spectra, the furocoumarin skeleton atoms numeration system given in structure **14** was used. The IR spectra were recorded by means of the KBr pellet technique on a Bruker Vector-22 spectrometer. The UV spectra were obtained on an HP 8453 UV-Vis spectrometer (Hewlett-Packard, Waldbronn, Germany). The melting points were determined on a Stuart SMF-38 melting point apparatus (Bibby Scientific, Staffordshire, UK) and are uncorrected. Elemental analysis was carried out on a Carlo-Erba 1106 Elemental analysis instrument (Carlo-Erba, Milan, Italy). Spectral and analytical investigations were carried out at Collective Chemical Service center of Siberian Branch of the Russian Academy of Sciences.

Reaction products were isolated by column chromatography on silica gel 60 (0.063–0.200 mm, Merck KGaA, Darmstadt, Germany) and eluted with chloroform and chloroform-ethanol (100:1; to 25:l). The reaction progress and the purity of the obtained compounds were monitored by TLC on Silufol UV-254 plates (Kavalier, Czech Republic, CHCl_3_-EtOH, 25:1; detection under UV light or by treatment with iodine vapor).

Chemicals used—TsCl, Ti(O-*i*Pr)_4_, n-BuLi, CuI, PPh_3_, Lindlar’s catalysts and (trimethylsilyl)acetylene **30**—were purchased from Sigma-Aldrich (St. Louis, MO, USA) or Alfa Aesar (GmbH, Karlsruhe, Germany). Dichlorobis(triphenylphosphine)palladium(II) was obtained as described in [31]. 1-Ethynyl-3,4,5-trimethoxybenzene (**21a**) [32], 1-ethynyl-3-hydroxy-4-trimethoxybenzene (**21b**) [33], 1-ethynyl-2,3,4-trimethoxybenzene (**21c**) [34], 1-ethynyl-3,4-dihydroxybenzene (**21d**) [35], 1-ethynyl-2-hydroxy-3-methoxy-5-formylbenzene (**21e**) [36], 3,4-dihydroxybromobenzene (**33**) [37] and combretastatin A-4 (**1**) [22] are known compounds and were prepared by the reported methods. Solvents (THF, benzene, MeOH) and Et_3_N were purified by standard methods and distilled in a stream of argon just before use.

### 3.2. Synthesis and Spectral Data

*2-Isopropyl-2-[(3,4,5-trimethoxyphenyl)ethynyl]-2H-furo[3,2-g]chromene-3,7-dione* (**16**). (*a*) To a solution of oreoselone (**14**, 488 mg, 2 mmol) in THF (7 mL) under argon was added TsCl (470 mg, 2.5 mmol) and Pd(PPh_3_)_2_Cl_2_ (7 mg, 0.1 mmol). The mixture was heated at 60 °C for 6 h (TLC), then 1-ethynyl-3,4,5-trimethoxybenzene (**21a**, 770 mg, 4 mmol) and Et_3_N (0.84 mL, 6 mmol) was added. The reaction mixture was heated under stirring another 7 h. After cooling, 3 mL of water was added and the mixture was extracted with methylene chloride (5 × 4 mL). The combined extract was washed with water, dried over MgSO_4_ and filtered. The solvent was removed under reduced pressure, and the residue was subjected to column chromatography to isolate 538 mg (62%) of **16** and 40 mg (12%) of **22**. Compound **16** was recrystallized from diethyl ether, m.p. 101–102 °С. IR (KBr, *ν*, cm^−^^1^): 2974, 2927, 2879, 2852, 2150, 1732, 1664, 1626, 1531, 1481, 1411, 1390, 1353, 1286, 1253, 1126, 1095, 1039, 970, 948, 916, 866, 827, 734, 661. ^1^H-NMR (600 MHz, CDCl_3_, δ_H_): 0.98 (d, *J =* 6.9 Hz, 3Н, СН_3_), 1.28 (d, *J* = 6.9 Hz, 3Н, СН_3_), 2.46 [m, 1Н, СН-(СН_3_)_2_], 3.84 (s, 9Н, 3 × OСН_3_), 6.34 (d, *J* = 9.6 Hz, 1Н, Н-6), 6.96 (s, 1Н, Н-9), 7.04 (s, 2Н, Н-2',6'), 7.68 (d, *J =* 9.6 Hz, 1Н, Н-5), 7.87 (s, 1Н, Н-4). ^13^C-NMR (150 MHz, CDCl_3_, δ_C_): 16.5 (CH_3_), 17.4 (CH_3_), 36.4 (CH), 56.0 (3C-CH_3_), 72.1 (C-1a), 82.6 (C-1b), 99 (C-2), 101.4 (C-9), 106.5 (C-2' and C-6'), 115.2 (C-6), 115.6, 115.5 (C-3a,4a),119.4 (C-1'), 125.8 (C-4), 131.5 (C-4'), 143.0 (C-5), 153.4 (C-8a), 155.8 (C-3' and C-5'), 158.6 (C-7), 169.4 (C-9a), 193.8 (C-3). UV (EtOH) λ_max_, (lgε): 257 (4.56), 297 (4.3), 307 (4.27), 348 (4.11) nm. Anal. Calcd for C_25_H_22_O_7_: С, 69.12; Н, 5.10; found С, 69.29; Н, 5.02. (*b*) A mixture of 2-(*p*-toluenesulfonyl)oreoselone (**15**, 398 mg, 1 mmol), 1-ethynyl-3,4,5-trimethoxybenzene (**21a**, 385 mg, 2 mmol), Et_3_N (0.42 mL, 3 mmol), and Pd(PPh_3_)_2_Cl_2_ (7 mg, 0.1 mmol) in 5 mL of anhydrous THF was heated for 7 h under reflux in argon. The mixture was evaporated, the residue was treated with 10 mL of water and extracted with methylene chloride (4 × 5 mL). The combined extract was dried over MgSO_4_, filtered and evaporated. The residue was subjected to column chromatography on silica gel to isolate 287 mg (66%) of compound **16** and 12 mg (5%) of compound **22**.

*2-[(3-Hydroxy-4-methoxyphenyl)ethynyl]-2-isopropyl-2H-furo[3,2-g]chromene-3,7-dione* (**17**)*.* This compound (374 mg, 48%) was prepared as a yellow oil from oreoselone (**14**, 488 mg, 2 mmol) and 1-ethynyl-3-hydroxy-4-methoxybenzene (**21b** (592 mg, 4 mmol) using the procedure (*a*) described for **16**. IR (KBr, *ν*, cm^−^^1^): 3467, 3086, 3062, 3030, 2976, 2923, 2850, 2191, 1751, 1660, 1625, 1600, 1494, 1419, 1353, 1267, 1207, 1149, 1110, 1066, 1028, 977, 935, 806, 752, 696. ^1^H-NMR (400 MHz, CDCl_3_, δ_H_): 1.09 (d, *J* = 6.9 Hz, 3Н, СН_3_), 1.20 (d, *J =* 6.9 Hz, 3Н, СН_3_), 3.03 [m, 1Н, СН-(СН_3_)_2_], 3.79 (s, 3Н, OСН_3_), 6.05 (s, 1H, OH), 6.40 (d, *J* = 9.6 Hz, 1Н, Н-6), 6.92 (d, *J* = 7.8 Hz, 1Н, Н-5'), 6.97 (dd, *J* = 7.8 and 1.8 Hz, 1Н, Н-6'), 6.98 (s, 1Н, Н-9), 6.99 (d, *J* = 1.8 Hz, 1Н, Н-2'), 7.66 (d, *J* = 9.6 Hz, 1Н, Н-5), 7.85 (s, 1Н, Н-4). ^13^C-NMR (100 MHz, CDCl_3_, δ_C_): 16.2 (CH_3_), 17.4 (CH_3_), 30.1 (CH), 54.1 (CH_3_), 73.5 (C-1a), 82.4 (C-1b), 96.1 (C-2), 100.1 (C-9), 112.4 (C-6), 113.1 (C-5'), 113.4 (C-3a), 115.2 (C-4a), 118.1 (C-1'), 120.5 (C-2'), 123.1 (C-6'), 125.7 (C-4), 142.3 (C-5), 147.9 (C-3'), 149.1 (C-4'), 156.6 (C-8a), 158.1 (C-7), 172.6 (C-9a), 195.5 (C-3). UV (EtOH) λ_max_ (lgε): 258 (4.52), 295 (3.96), 307 (3.88), 351 (3.93) nm. Anal. Calcd for C_23_H_18_O_6_: С, 70.76; Н, 4.65; found С, 70.39; Н, 4.92.

*2-Isopropyl-2-[(2,3,4-trimethoxyphenyl)ethynyl]-2H-furo[3,2-g]chromene-3,7-dione* (**18**)*.* Compound **18** (560 mg, 65%) was prepared from oreoselone (**14**, 488 mg, 2 mmol) and 1-ethynyl-2,3,4-trimethoxybenzene (**21c**, 770 mg, 4 mmol) using the procedure (*a*) described for **16**. Compound **22** (30 mg, 10%) was also isolated. Compound **18**, m.p. 101–102 °С (ether). IR (KBr, *ν*, cm^−^^1^): 3066, 2976, 2939, 2861, 2189, 1737, 1679, 1625, 1487, 1423, 1388, 1352, 1286, 1224, 1141, 1122, 1093, 1022, 983, 943, 866, 825, 790, 734, 691. ^1^H-NMR (400 MHz, CDCl_3_, δ_H_): 0.91 (d, *J* = 6.9 Hz, 3Н, СН_3_), 1.31 (d, *J =* 6.9 Hz, 3Н, СН_3_), 2.50 [m, 1Н, СН-(СН_3_)_2_], 3.82 (s, 3Н, OСН_3_), 3.88 (s, 3Н, OСН_3_), 3.97 (s, 3Н, OСН_3_), 6.34 (d, *J* = 9.6 Hz, 1Н, Н-6), 6.70 (d, *J* = 8.2 Hz, 1Н, Н-5'), 6.99 (s, 1Н, Н-9), 7.52 (d, 1Н, *J* = 8.2 Hz, Н-6'), 7.72 (d, 1Н, *J* = 9.6 Hz, Н-5), 7.90 (1Н, s, Н-4). ^13^C-NMR (100 MHz, CDCl_3_, δ_C_): 16.6 (CH_3_), 17.5 (CH_3_), 36.4 (CH), 56.0 (CH_3_), 60.8 (CH_3_), 62.2 (CH_3_), 71.6 (C-1a), 82.3 (C-1b), 98.9 (C-2), 101.5 (C-9), 107.2 (C-5'), 115.4 (C-3a,4a), 115.3 (C-6), 115.6, 123.1 (C-1'), 124.0 (C-6'), 125.9 (C-4), 141.4 (C-3'), 143.1 (C-5), 156.7 (C-2'), 158.8 (C-4'), 159.1 (C-8a),161.1 (C-7), 169.5 (C-9a), 192.6 (C-3). UV (EtOH) λ_max_ (lgε): 257 (4.42), 297 (4.17), 307 (4.14), 348 (3.97) nm. Anal. Calcd for C_25_H_22_O_7_: С, 69.12; Н, 5.10; found С, 69.49; Н, 5.12.

*2-[(3,4-Dihydroxyphenyl)ethynyl]-2-isopropyl-2H-furo[3,2-g]chromene-3,7-dione* (**19**)*.* Compound **19** (330 mg, 44%) was prepared from oreoselone (**14**, 488 mg, 2 mmol) and 1-ethynyl-2,3,4-trimethoxybenzene (**21d**, 540 mg, 4 mmol) using the procedure (*a*) described for **16**. Compound **22** (82 mg, 25%) was also isolated. Compound **19**, m.p. 84–86 °С (ether). IR (KBr, *ν*, cm^−^^1^): 3340, 3320, 3087, 3071, 2974, 2929, 2879, 2852, 2114, 1732, 1664, 1625, 1595, 1531, 1390, 1353, 1286, 1253, 1126, 1095, 1039, 948, 866, 827, 735. ^1^H-NMR (400 MHz, CDCl_3_, δ_H_): 0.96 (d, *J* = 7.0 Hz, 3Н, СН_3_), 1.37 (d, *J =* 7.0 Hz, 3Н, СН_3_), 2.55 [m, 1Н, СН-(СН_3_)_2_], 6.40 (d, *J* = 9.6 Hz, 1Н, Н-6), 7.05 (s, 1Н, Н-9), 7.46 (d, *J* 1.8 Hz, 1Н, Н-2'), 7.52 (dd, *J* = 8.0 and 1.8 Hz, 1Н, Н-6'), 7.66 (d, *J* = 8.0 Hz, 1Н, Н-5'), 7.73 (d, *J* = 9.6 Hz, 1Н, Н-5), 7.93 (s, 1Н, Н-4). ^13^C-NMR (100 MHz, CDCl_3_): 15.6 (CH_3_), 16.0 (CH_3_), 31.1 (CH), 73.9 (C-1a), 81.9 (C-1b), 98.6 (C-2), 101.4 (C-9), 115.7 (C-3a), 116.0 (C-6), 116.7 (C-4a), 117.8 (C-1'), 120.2 (C-5'), 122.7 (C-2'), 124.1 (C-4), 126.1 (C-6'), 143.3 (C-5), 158.5 (C-8a), 159.3 (C-3'), 160.4 (C-4'), 161.9 (C-7), 171.8 (C-9a), 191.9 (C-3). UV (EtOH) λ_max_ (lgε): 256 (4.4), 303 (3.92), 348 (3.87) nm. Anal. Calcd for C_22_H_16_O_6_: С, 70.21; Н, 4.29; found С, 70.49; Н, 4.20.

*2-[(5-Formyl-3-hydroxy-3-methoxyphenyl)ethynyl]-2-isopropyl-2H-furo[3,2-g]chromene-3,7-dione* (**20**). Compound **20** (493 mg, 59%) was prepared from oreoselone (**14**, 488 mg, 2 mmol) and 1-ethynyl-5-formyl-2-hydroxy-3-methoxybenzene (**21e**, 705 mg, 4 mmol) using the procedure (*a*) described for **16**, or from 2-(*p*-toluenesulfonyl)oreoselone (**15**, 398 mg, 1 mmol) and 1-ethynyl-5-formyl-2-hydroxy-3-methoxybenzene (**21e**, 353 mg, 2 mmol) using the procedure (*b*) described for **16**. Yield 60%. Compound **22** was also isolated [62 mg, procedure (a) and 23 mg, procedure (b)]. Compound **20**, m.p. 91–93 °С (ether). IR (KBr, *ν*, cm^−^^1^): 3456, 3155, 3065, 2978, 2939, 2881, 2839, 2112, 1738, 1680, 1626, 1582, 1554, 1487, 1423, 1389, 1352, 1286, 1260, 1250, 1225, 1201, 1141, 1122, 1094, 1043, 1022, 984, 970, 943, 916, 866, 826, 790, 760, 735, 690, 6800. ^1^H-NMR (400 MHz, CDCl_3_, δ_H_): 0.91 (d, *J* = 6.9 Hz, 3Н, СН_3_), 1.29 (d, *J =*6.9 Hz, 3Н, СН_3_), 2.48 [m, 1Н, СН-(СН_3_)_2_], 3.77 (s, 3Н, OСН_3_), 6.32 (d, *J* = 9.8 Hz, 1Н, Н-6), 6.96 (s, 1Н, Н-9), 7.43 (br s, 1Н, Н-4'), 7.50 (br s, 1H, OH), 7.60 (br s, 1Н, Н-6'), 7.69 (d, *J* = 9.8Hz, 1Н, Н-5), 7.88 (s, 1Н, Н-4), 9.91 (s, 1H, CHO). ^13^C-NMR (100 MHz, CDCl_3_, δ_C_): 15.2 (CH_3_), 15.6 (CH_3_), 33.7 (CH), 55.4 (OCH_3_), 71.3 (C-1a), 80.2 (C-1b), 98.3 (C-2), 101.1 (C-9), 115.4 (C-3a), 115.7 (C-6), 116.4 (C-4'), 118.3 (C-4a), 119.5 (C-1'), 121.5 (C-6'), 125.7 (C-4), 133.6 (C-5'), 143.1 (C-5), 156.6 (C-3'), 158.9 (C-2'), 161.6 (C-7), 165.1 (C-8a), 171.5 (C-9a), 191.6 (CHO), 192.3 (C-3). UV (EtOH) λ_max_ (lgε): 253 (4.21), 328 (3.45), 350 (3.15) nm. Anal. Calcd for C_24_H_18_O_7_: С, 68.90; Н, 4.34; found С, 68.69; Н, 4.56.

*(Z)-2-Isopropyl-2-(3,4,5-trimethoxystyryl)-2H-furo[3,2-g]chromene-3,7-dione* (**2**)*.* A solution of *n*-BuLi (0.08 mL, 1.04 mmol) was added dropwise to a solution of 2-isopropyl-2-[(3,4,5-trimethoxyphenyl)ethynyl]-2*H*-furo[3,2-*g*]chromene-3,7-dione (**16**, 100 mg, 0.23 mmol) and tetraisopropoxytitanium (140 mg, 0.52 mmol) in anhydrous THF (3 mL) at −78 °C. The stirring was continued for 10 min at the same temperature. The reaction mixture was warmed to room temperature and heated at 50 °С for 2 h. After cooling, the reaction was quenched with a saturated solution of NH_4_Cl (3 mL), water (3 mL), and extracted with dichloromethane (3 × 4 mL), the combined organic layers was washed with water, dried over anhydrous MgSO_4_, and filtered. The solvent was evaporated, and the residue was subjected to column chromatography on silica gel (chloroform and chloroform–ethanol 100:1 as eluent) to afford compound **2** (42 mg, 42% yield) as a yellow powder, m.p. 91–92 °С (ether). IR (KBr, *ν*, cm^−^^1^): 3047, 2974, 2935, 1736, 1701, 1628, 1585, 1474, 1390, 1356, 1286, 1226, 1195, 1120, 1034, 1011, 972, 900, 866, 840, 827, 756, 740, 700, 681. ^1^H-NMR (400 MHz, CDCl_3_, δ_H_): 0.88 (d, *J =* 6.9 Hz, 3Н, СН_3_], 1.11 (d, *J =*6.9 Hz, 3Н, СН_3_), 3.20 [m, 1Н,СН-(СН_3_)_2_], 3.83 (s, 9Н, 3 × OСН_3_), 6.23 (d, *J* = 9.2 Hz, 1Н, Н-1a), 6.36 (d, *J* = 9.7 Hz, 1Н, Н-6), 6.74 (d, *J* = 9.2 Hz, 1Н, Н-1b), 6.94 (s, 1Н, Н-9), 7.10 (br s, 2Н, Н-2',6'), 7.67 (d, *J* = 9.7 Hz, 1Н, Н-5), 7.90 (s, 1Н, Н-4). ^13^C-NMR (100 MHz, CDCl_3_, δ_C_): 16.1 (CH_3_), 17.0 (CH_3_), 29.2 (CH), 55.6 (3×OCH_3_), 98.3 (C-2), 101.0 (C-9), 106.1 (C-2',6'), 114.8 (C-3a,4a), 115.1, 115.2 (C-6), 125.4 (C-4), 119.0 (C-1'), 126.8 (C-1a), 131.1 (C-1b), 131.8 (C-4'), 142.6 (C-5), 153.0 (C-8a), 155.3 (C-3',5'), 158.2 (C-7), 168.9 (C-9a), 192.4 (C-3). UV (EtOH) λ_max_ (lgε): 255 (4.44), 296 (4.07), 310 (4.01), 344 (sh), 354 (3.9) nm. Anal. Calcd for C_25_H_24_O_7_: С, 68.80; Н, 5.54; found С, 68.69; Н, 5.17.

*(Z)-2-(3-Hydroxy-4-methoxystyryl)-2-isopropyl-2H-furo[3,2-g]chromene-3,7-dione* (**3**). Lindlar’s catalyst (5 mg, 2 mol %) was added to a solution of 2-isopropyl-2-[(3-hydroxy-4-methoxy-phenyl)ethynyl]-2*H*-furo[3,2-*g*]chromene-3,7-dione (**17**, 100 mg, 0.26 mmol) in dry ethanol (6 mL). The system was filled with hydrogen, and the reaction mixture was stirred in a hydrogen flow for 20 h and then concentrated under reduced pressure. Column chromatography on silica gel afforded compound **3** (31 mg, 32% yield) as a yellow oil. IR (KBr, *ν*, cm^−^^1^): 3520, 3488, 3398, 2960, 2925, 2852, 1750, 1726, 1724, 1630, 1576, 1510, 1480, 1463, 1356, 1335, 1300, 1250, 1176, 1157, 1143, 1105, 1032, 978, 935, 901, 885, 831, 813, 760, 744, 700, 671. ^1^H-NMR δ_H_ (600 MHz, CDCl_3_, δ_H_): 0.87 [d, *J =* 6.9 Hz, 3Н, СН_3_), 1.15 [d, *J* = 6.9 Hz, 3Н, СН_3_), 2.37 [m, 1Н, СН-(СН_3_)_2_], 3.76 (s, 3Н, OCH_3_), 6.26 (d, *J* = 9.5 Нz, 1Н, Н-1b), 6.33 (d, *J* = 9.7 Нz, 1Н, Н-6), 6.92 (d, *J* = 7.8 Нz, 1Н, Н-5'), 6.79 (d, *J* 9.5 Нz, 1Н, Н-1b), 6.89 (dd, *J* = 8.0 and 1.8 Нz, 1Н, Н-6'), 7.00 (s, 1Н,Н-9), 7.06 (d, *J* = 8.0 Нz, 1Н, Н-5'), 7.25 (d, *J* = 1.8 Нz, 1Н, Н-2'), 7.68 (d, *J* = 9.7 Нz, 1Н, Н-5), 7.78 (s, 1Н, Н-4). ^13^C-NMR (150 MHz, CDCl_3_, δ_C_): 15.6 (CH_3_), 18.6 (CH_3_), 31.2 (CH), 55.3 (OCH_3_), 92.9 (C-2), 100.9 (C-9), 113.6 (C-6), 114.4 (C-5'), 114.8 (C-3a), 116.4 (C-4a), 119.3 (C-2'), 121.7 (C-6'), 124.3 (C-4), 125.2 (C-1a), 129.2 (C-1b), 134.9 (C-1'), 143.5 (C-5), 149.2 (C-3'), 150.3 (C-4'), 159.4 (C-8a), 160.1 (C-7), 173.9 (C-9a),199.4 (C-3). UV (EtOH) λ_max_ (lgε): 253 (4.41), 296 (4.08), 308 (4.06), 341 (sh), 353 (4.1) nm, Anal. Calcd for C_23_H_20_O_6_: С, 70.40; Н, 5.14; found С, 70.78; Н, 4.92. 

*(Z)-2-Isopropyl-2-(2,3,4-trimethoxystyryl)-2H-furo[3,2-g]chromene-3,7-dione* (**6**). Compound **6** (40 mg) was prepared from 2-isopropyl-2-[(2,3,4-trimethoxyphenyl)ethynyl]-2*H*-furo[3,2-*g*]chromene-3,7-dione (**18**, 100 mg, 0.23 mmol) using the procedure described for **2**. Yield 40%, m.p. 112–114 °С (ether). IR (KBr, *ν*, cm^−^^1^): 3047, 2974, 1736, 1701, 1628, 1585, 1473, 1391, 1355, 1286, 1220, 1196, 1121, 1034, 1011, 972, 900, 866, 850, 827, 800, 756, 740, 680. ^1^H-NMR (400 MHz, CDCl_3_, δ_H_): 0.98 (d, *J* = 6.9 Нz, 3Н, СН_3_), 1.19 (3Н, *J* = 6.9 Нz, d, СН_3_), 3.28 [m, 1Н, СН-(СН_3_)_2_], 3.91 (s, 3Н, OСН_3_), 3.92 (s, 3Н,OСН_3_), 3.94 (s, 3Н,OСН_3_), 6.23 (d, *J* = 9.3 Нz, 1Н, Н-1a), 6.45 (d, *J* = 9.6 Нz, 1Н, Н-6), 6.67 (d, *J* = 8.3 Нz, 1Н, Н-5'), 6.81 (d, *J* = 9.3 Нz, 1Н, Н-1b), 7.18 (d, *J =* 8.3 Нz, 1Н, Н-6'), 7.97 (d, *J* = 9.6 Нz, 1Н, Н-5), 8.19 (s, 1Н, Н-4). ^13^C-NMR (100 MHz, CDCl_3_, δ_C_): 16.5 (CH_3_), 17.4 (CH_3_), 31.6 (CH), 55.9 (OCH_3_), 62.1 (OCH_3_), 60.7 (OCH_3_), 99.5 (C-2), 101.4 (C-9), 107.2 (C-5'), 115.2 (C-6), 115.5, 115.6 (C-3a, 4a), 123.9 (C-6'), 123.0 (C-1'), 125.9 (C-4), 126.9 (C-1a), 133.1 (C-1b), 141.3 (C-5), 144.2 (C-3'), 156.6 (C-2'), 158.7 (C-4'), 159.0 (C-8a), 160.9 (C-7), 170.7 (C-9a), 190.5 (C-3). UV (EtOH) λ_max_ (lgε): 255 (4.32), 296 (3.94), 310 (3.88), 344 (sh), 354 (3.78) nm. Anal. Calcd for C_25_H_24_O_7_: С, 68.80; Н, 5.54; found С, 69.09; Н, 5.42.

*(Z)-2-[(3,4-Dihydroxystyryl)-2-isopropyl-2H-furo[3,2-g]chromene-3,7-dione* (**7**)*.* Compound **7** (24 mg) was prepared by partial reduction of 2-[(3,4-dihydroxyphenyl)ethynyl]-2-isopropyl-2*H*-furo[3,2-*g*]chromene-3,7-dione (**19**, 100 mg, 0.26 mmol) using the procedure described for **3** (reaction time 30 h). Yield 24%, m.p. 68–71 °С (ether). IR (KBr, *ν*, cm^−^^1^): 3470, 2979, 2922, 2851, 1751, 1661, 1626, 1601, 1598, 1495, 1420, 1354, 1327, 1267, 1207, 1150, 1111, 1086, 1028, 1002, 978, 935, 914, 823, 800, 752, 696. ^1^H-NMR (400 MHz, CDCl_3_, δ_H_): 0.85 (d, *J* = 6.9 Нz, 3Н, СН_3_), 0.91 [d, *J =* 6.9 Нz, 3Н, СН_3_), 2.99 [m, 1Н, СН-(СН_3_)_2_], 6.21 (d, *J* = 9.5 Нz, 1Н, Н-1a), 6.37 (d, *J* = 9.7 Нz, 1Н, Н-6), 6.37 (d, *J =* 8.3 Нz, 1Н, Н-5'), 6.81 (d, *J* = 9.5 Нz, 1Н, Н-1b), 6.89 (s, 1Н, Н-9), 7.36 (d, *J* = 1.8 Нz, 1Н, Н-2'), 7.49 (dd, *J* = 8.0 and 1.8 Нz, 1Н, Н-6'), 7.65 (d, *J* = 8.0 Нz, 1Н, Н-5'), 7.91 (d, *J =* 9.6 Нz, 1Н, Н-5), 8.03 (s, 1Н, Н-4).^ 13^C-NMR (100 MHz, CDCl_3_, δ_C_): 15.1 (CH_3_), 15.5 (CH_3_), 30.5 (CH), 98.5 (C-2), 100.9 (C-9), 115.2 (C-3a), 115.5 (C-6), 116.2 (C-5'), 117.2 (C-4a), 119.7 (C-1'), 122.2 (C-2'), 123.6 (C-6'), 125.5 (C-4), 127.1 (C-1a), 132.4 (C-1b), 142.8 (C-5), 157.9 (C-3'), 158.8 (C-4'), 159.9 (C-8a), 161.4 (C-7), 171.3 (C-9a), 190.3 (C-3). UV (EtOH) λ_max_ (lgε): 256 (4.76), 292 (4.33), 349 (4.13) nm. Anal. Calcd for C_22_H_18_O_6_: С, 69.83; Н, 4.79; found С, 70.10; Н, 4.31. 

*(Z)-2-(5-Formyl-3-hydroxy-3-methoxystyryl)-2-isopropyl-2H-furo[3,2-g]chromene-3,7-dione* (**8**). Compound **8** (37 mg) was prepared from 2-[(5-formyl-3-hydroxy-3-methoxyphenyl)ethynyl]-2-isopropyl-2*H*-furo[3,2-*g*] chromene-3,7-dione (**20**, 100 mg, 0.24 mmol) using the procedure described for **3**. Yield 37%, a yellow oil. IR (KBr, *ν*, cm^−^^1^): 3435, 3063, 3042, 2960, 2925, 2853, 2808, 1739, 1726, 1714, 1629, 1600, 1576, 1510, 1500, 1464, 1440, 1394, 1355, 1334, 1300, 1250, 1230, 1176, 1157, 1144, 1105, 1032, 978, 935, 915, 893, 831, 814, 761, 744, 720, 668. ^1^H-NMR (400 MHz, CDCl_3_, δ_H_): 0.97 (d, *J* = 6.9 Нz, 3Н, СН_3_], 1.07 [d, *J* = 6.9 Нz, 3Н, СН_3_), 3.15 [m, 1Н, СН-(СН_3_)_2_], 3.92 (s, 3Н, OСН_3_), 6.23 (d, *J* = 9.3 Нz, 1Н, Н-1a), 6.37 (1Н, *J =* 9.8 Нz, d, Н-6), 6.41 (d, *J* = 1.8 Нz, 1Н, Н-6'), 6.83 (d, *J* = 9.3 Нz, 1Н, Н-1b), 7.05 (s, 1Н, Н-9), 7.69 (d, 1Н, *J* = 9.6 Нz, Н-5), 7.78 (1Н, *J* = 1.8 Нz, d, Н-4'), 7.85 (s, 1Н, Н-4), 8.07 (1Н, *J =* 8.8 Нz, d, Н-1b), 9.98 (s, 1Н, CНO). ^13^C-NMR (100 MHz, CDCl_3_, δ_C_): 15.6 (CH_3_), 15.9 (CH_3_), 34.0 (CH), 55.7 (OCH_3_), 99.5 (C-2), 100.6 (C-9), 115.6 (C-6), 116.0 (C-3a), 116.7 (C-4a), 118.7 (C-4'), 121.8 (C-6'), 119.8 (C-1'), 126.0 (C-4), 127.5 (C-1a), 132.6 (C-1b), 133.9 (C-5'), 144.8 (C-5), 156.9 (C-3'), 158.9 (C-2'), 161.9 (C-8a), 165.3 (C-7), 171.8 (C-9a), 191.9 (СНО), 194.6 (C-3). UV (EtOH) λ_max_ (lgε): 256 (4.41), 291 (3.97), 349 (3.78) nm. Anal. Calcd for C_24_H_20_O_7_: С, 68.57; Н, 4.80; found С, 68.24; Н, 4.68.

*2-Isopropyl-3-[(2,3,4-trimethoxyphenyl)ethynyl]-7H-furo[3,2-g]chromene-7-one* (**25**). To a solution of 2-isopropyl-3-(trifluoromethanesulfonyloxy)psoralene (**23**, 150 mg, 0.4 mmol) and 1-ethynyl-2,3,4-trimethoxybenzene (**21c**, 85 mg, 0.44 mmol) in benzene (5 mL) was added CuI (1.5 mg, 2 mol %), Pd(PPh_3_)_2_Cl_2_ (11 mg, 4 mol %), and Et_3_N (0.076 mL, 0.55 mmol; 1.4 equiv) under argon. The reaction mixture was stirred at 80 °C for 8 h (TLC). The mixture was cooled and 5 mL of water was added. The separated water layer was extracted with methylene chloride (5 × 4 mL). The combined organic extracts was washed with water, dried over MgSO_4_, filtered, and concentrated under reduced pressure. The residue was subjected to column chromatography on silica gel. Eluting with chloroform and crystallization from diethyl ether gave 122 mg (73%) of compound **25**. M.p. 121–122 °С (ether). IR (KBr, *ν*, cm^−^^1^): 3051, 2979, 2927, 2854, 2472, 2118, 1732, 1622, 1603, 1580, 1514, 1470, 1386, 1363, 1321, 1277, 1252, 1213, 1194, 1167, 1140, 1115, 1096, 1068, 1043, 980, 959, 935, 916, 870, 850, 820, 770, 750, 741, 702, 677. ^1^H-NMR (400 MHz, CDCl_3_, δ_H_): 1.35 (d, *J* = 7.0 Hz, 3Н, СН_3_), 1.37 [d, *J* = 7.0 Hz, 3Н, СН_3_), 3.25 [m, 1Н, СН-(СН_3_)_2_], 3.86 (s, 3Н, OСН_3_), 3.92 (s, 3Н, OСН_3_), 4.01 (s, 3Н, OСН_3_), 6.40 (d, *J* = 9.8 Hz, 1Н, Н-6), 6.72 (d, *J* = 8.2 Hz, 1Н, Н-5'), 6.98 (s, 1Н, Н-9), 7.59 (d, *J* = 8.2 Hz, 1Н, Н-6'), 7.79 (s, 1Н, Н-4), 7.80 (d, 1Н, *J* = 9.8 Hz, Н-5). ^13^C-NMR (100 MHz, CDCl_3_, δ_C_): 20.3 (CH_3_), 20.5 (CH_3_), 25.9 (CH), 56.1 (OCH_3_), 60.9 (OCH_3_), 62.3 (OCH_3_), 86.5 (C-1a), 94.0 (C-1b), 95.5 (C-3), 97.5 (C-9), 107.3 (C-5'), 100.5 (C-4a), 111.1 (C-6), 116.6 (C-3a), 118.9 (C-1'), 124.2 (C-6'), 126.5 (C-4), 141.6 (C-3'), 143.5 (C-5), 152.0 (C-9a), 153.1 (C-8a), 154.1, 154.7 (C-2',4'), 156.9 (C-2), 159.2 (C-7). UV (EtOH) λ_max_ (lgε): 249 (3.65), 306 (3.75), 327 (3.73), 351 (sh), 355(2.85) nm. Anal. Calcd for C_25_H_22_O_6_: С, 71.76; Н, 5.30; found С, 71.49; Н, 5.12.

*3-[(3,4-Dihydroxyphenyl)ethynyl]-2-isopropyl-7H-furo[3,2-g]chromene-7-one* (**26**)*.* Compound **26** was prepared by two methods. Reaction of psoralene derivative **23** (150 mg, 0.4 mmol) with 1-ethynyl-3,4-dihydroxybenzene (**21d**, 60 mg, 0.44 mmol) by the procedure described for **25** (method *a*) gave 98 mg (68%) of compound **26**. (*b*) To a solution of 3-ethynyl-2-isopropylpsoralene (**32**, 100 mg, 0.4 mmol) in benzene (5 mL) was added 3,4-dihydroxybromobenzene (**33**, 83 mg, 0.44 mmol), 1.5 mg (2 mol %) of CuI, 11 mg (4 mol %) of Pd(PPh_3_)_2_Cl_2_, and 0.076 mL (1.4 equiv, 0.55 mmol) of Et_3_N under argon. The mixture was stirred at 80 °C for 10 h (TLC). Then the solution was cooled, 3 mL of water was added and reaction mixture was extracted with methylene chloride (5 × 4 mL). The combined extract was washed with water, dried over MgSO_4_, filtered, and concentrated under reduced pressure. The residue was subjected to column chromatography on silica gel. Eluting with chloroform and crystallization from diethyl ether gave 60 mg (42%) of compound **26**. M.p. 104–105 °С. IR (KBr, *ν*, cm^−^^1^): 3450, 3327, 3051, 2980, 2880, 2556, 2472, 2096, 1732, 1716, 1697, 1620, 1590, 1550, 1514, 1431, 1364, 1278, 1253, 1213, 1191, 1160, 1140, 1095, 1041, 980, 950, 876, 860, 849, 825, 770, 754, 740, 685, 650. ^1^H-NMR (400 MHz, CDCl_3_, δ_H_): 1.38 (d, *J* = 7.0 Hz, 3Н, СН_3_), 1.40 (d, *J* = 7.0 Hz, 3Н, СН_3_), 3.30 [m, 1Н, СН-(СН_3_)_2_], 4.09 (br.s, 2Н, OH), 6.32 (d, *J* = 9.7 Hz, 1Н, Н-6), 7.07 (s, 1Н, Н-9), 7.26 (d, *J* = 2.0 Hz, 1Н, Н-2'), 7.32 (dd, *J* = 8.0 and 2.0 Hz, 1Н, Н-6'), 7.46 (d, *J* = 8.0 Hz, 1Н, Н-5'), 7.77 (d, *J* = 9.7 Hz, 1Н, Н-5), 7.80 (s, 1Н, Н-4). ^13^C-NMR (100 MHz, CDCl_3_, δ_C_): 20.0 (CH_3_), 20.1 (CH_3_), 25.8 (CH), 86.4 (C-1a), 94.0 (C-1b), 100.3 (C-3), 102.8 (C-9), 112.3 (C-4a), 115.2 (C-6), 115.3 (C-3a), 116.5 (C-1'), 120.2 (C-5'), 121.2 (C-4), 124.5 (C-2'), 126.2 (C-6'), 144.2 (C-5), 145.9 (C-3'), 147.4 (C-4'), 152.1 (C-9a), 155.3 (C-8a), 160.4 (C-2), 161.3 (C-7). UV (EtOH) λ_max_ (lgε): 252 (3.64), 309 (3.94), 339 (3.43), 355 (sh) nm. Anal. Calcd for C_22_H_16_O_5_: С, 73.33; Н, 4.48; found С, 73.30; Н, 4.55.

*3-[(5-Formyl-3-hydroxy-3-methoxyphenyl)ethynyl]-2-isopropyl-7H-furo[3,2-g] chromene-7-one* (**27**). Compound **27** (112 mg) was prepared from psoralene derivative **23** (150 mg, 0.4 mmol) and 1-ethynyl-5-formyl-2-hydroxy-3-methoxybenzene (**21e**, 75 mg, 0.44 mmol) using the procedure described for **25**. Yield 70%, m.p. 110–112 °С (ether). IR (KBr, *ν*, cm^−^^1^): 3430, 3325, 2989, 2880, 2713, 2472, 2046, 1944, 1782, 1732, 1716, 1697, 1620, 1602, 1514, 1469, 1431, 1363, 1315, 1278, 1253, 1220, 1191, 1139, 1095, 1041, 1020, 980, 904, 860, 848, 769, 754, 695, 650, 600. ^1^H-NMR (400 MHz, CDCl_3_, δ_H_): 1.39 (d, *J* = 7.0 Hz, 3Н, СН_3_), 1.42 (d, *J* = 7.0 Hz, 3Н, СН_3_), 3.29 [m, 1Н, СН-(СН_3_)_2_], 3.98 (s, 3Н, OСН_3_), 6.36 (d, *J* = 9.8 Hz, 1Н, Н-6), 7.09 (s, 1Н, Н-9), 7.44 (br.s, 1Н, Н-4'), 7.50 (br.s, 1H, OH), 7.60 (br.s, 1Н, Н-6'), 7.75 (d, *J* = 9.8 Hz, 1Н, Н-5), 7.82 (s, 1Н, Н-4), 10.01 (s, 1H, CHO). ^13^C-NMR (100 MHz, CDCl_3_, δ_C_): 20.5 (CH_3_), 20.6 (CH_3_), 26.2 (CH), 60.6 (OCH_3_), 85.6 (C-1a), 91.0 (C-1b), 99.5 (C-3), 104.4 (C-9), 115.8 (C-3a), 116.3 (C-6), 116.9 (C-4'), 118.9 (C-4a), 119.8 (C-1'), 120.6 (C-6'), 128.7 (C-4), 132.2 (C-5'), 143.9 (C-5), 150.7 (C-3'), 152.5 (C-8a), 153.1 (C-9a), 154.6 (C-2'), 159.2 (C-2), 160.7 (C-7), 191.8 (CHO). UV (EtOH) λ_max_ (lgε): 246 (3.92), 305 (sh), 329 (3.63), 355 (2.81) nm. Anal. Calcd for C_24_H_18_O_6_: С, 71.64; Н, 4.51; found С, 71.30; Н, 4.58. 

*3-[(3,4-Dihydroxyphenyl)ethynyl]-2-isopropyl-9-[(4-methylpiperazin-1-yl)methyl]-7H-furo[3,2-g]-chromene-7-one* (**28**). Compound **28** (79 mg) was prepared from 2-isopropyl-9-((4-methylpiperazin-1-yl)methyl)-7-oxo-7*H*-furo[3,2-g]chromen-3-yl trifluoromethanesulfonate (**24**, 195 mg, 0.4 mmol) and 1-ethynyl-3,4-dihydroxybenzene (**21d**, 60 mg, 0.44 mmol) using the procedure described for **25**. Yield 42%, yellow powder, m.p. 118–120 °С (ether). IR (KBr, *ν*, cm^−^^1^): 3437, 2967, 2922, 2851, 2111, 1732, 1635, 1628, 1585, 1511, 1496, 1465, 1431, 1388, 1348, 1319, 1250, 1213, 1198, 1140, 1114, 1095, 1047, 957, 870, 820, 737, 700, 626, 602. ^1^H-NMR (400 MHz, CDCl_3_, δ_H_): 1.33 (d, *J* = 7.0 Hz, 3Н, СН_3_), 1.36 (3Н, d, *J* = 7.0 Hz, СН_3_), 2.32 (s, 3Н, NСН_3_), 2.38 (m, 4H, H-3'',5''), 2.68 (m, 4H, H-2'',6''), 3.25 [m, 1Н, СН-(СН_3_)_2_], 4.46 (d, 1H, *J* = 9.8 Hz, 1H, CH_2_), 4.58 (d, 1H, *J* = 9.8 Hz, 1H, CH_2_), 6.27 (d, *J* = 9.8 Hz, 1Н, Н-6), 6.52 (dd, *J* = 8.0 and 2.0 Hz, 1Н, Н-6'), 6.64 (d, *J* = 2.0 Hz, 1Н, Н-2'), 6.79 (d, *J* = 8.0 Hz, 1Н, Н-5'), 7.59 (d, *J* = 9.8 Hz, 1Н, Н-5), 7.74 (s, 1Н, Н-4), 8.00 (br.s, 2Н, OН). ^13^C-NMR (100 MHz, CDCl_3_, δ_C_): 20.1 (CH_3_), 20.3 (CH_3_), 25.9 (CH), 42.7 (NСН_3_), 48.5 (СН_2_N), 51.6 (C-2'',6''), 53.4 (C-3'',5''), 85.3 (C-1a), 90.7 (C-1b), 99.2 (C-3), 104.4 (C-9), 115.0 (C-1'), 115.6 (C-3a), 116.0 (C-6), 116.6 (C-4a), 117.6 (C-5'), 122.0 (C-2'), 128.4 (C-4), 130.8 (C-6'), 143.5 (C-5), 147.1 (C-2'), 148.7 (C-4'), 152.2 (C-8a), 153.0 (C-9a), 156.9 (C-2), 160.4 (C-7). UV (EtOH) λ_max_ (lgε): 250 (3.76), 284 (2.65), 327 (3.20), 346 (2.78) nm. Anal. Calcd for C_28_H_28_N_2_O_5_: С, 71.17; Н, 5.97; N, 5.93; found С, 70.91; Н, 6.02; N, 5.63.

*3-[(5-Formyl-3-hydroxy-3-methoxyphenyl)ethynyl]-2-isopropyl-9-[(4-methylpiperazin-1-yl)methyl]-7H-furo[3,2-g]chromen-7-one* (**29**). Compound **29** (119 mg) was prepared from 2-isopropyl-9-((4-methylpiperazin-1-yl)methyl)-7-oxo-7*H*-furo[3,2-*g*]chromen-3-yl trifluoromethanesulfonate (**24**, 195 mg, 0.4 mmol) and 1-ethynyl-5-formyl-2-hydroxy-3-methoxybenzene (**21e**, 5 mg, 0.44 mmol) using the procedure described for **25**. Yield 58%, m.p. 104–105 °С (ether). IR (KBr, *ν*, cm^−^^1^): 3402, 3117, 3080, 2958, 2850, 2783, 2711, 2611, 2172, 2133, 1726, 1693, 1610, 1593, 1537, 1464, 1427, 1402, 1367, 1331, 1304, 1252, 1229, 1213, 1180, 1142, 1107, 1065, 1030, 993, 968, 908, 876, 845, 752, 741, 715, 667, 633, 621. ^1^H-NMR (400 MHz, CDCl_3_, δ_H_): 1.36 (d, *J* = 7.0 Hz, 3Н, СН_3_), 1.39 (d, *J* = 7.0 Hz, 3Н, СН_3_), 2.38 (s, 3Н, NСН_3_), 2.42 (m, 4H, H-3'',5''), 2.65 (m, 4H, H-2'',6''), 3.25 [m, 1Н, СН-(СН_3_)_2_], 4.03 (s, 3Н, OСН_3_), 4.41 (d, *J* = 9.8 Hz, 1H, CH_2_), 4.48 (d, *J* = 9.8 Hz, 1H, CH_2_), 6.41 (d, *J* = 9.8 Hz, 1Н, Н-6), 6.89 (br.s, 1H, Н-4'), 7.41 (br.s, 1Н, OH), 7.44 (br.s, 1Н, Н-6'), 7.57 (s, 1Н, Н-4), 7.79 (d, *J* = 9.8 Hz, 1Н, Н-5), 9.99 (s, 1H, CHO). ^13^C-NMR (100 MHz, CDCl_3_, δ_C_): 20.2 (CH_3_), 20.5 (CH_3_), 25.9 (CH), 42.5 (NСН_3_), 48.2 (СН_2_N), 51.5 (C-2'',6''), 52.9 (C-3'',5''), 58.3 (OCH_3_), 85.2 (C-1a), 93.5 (C-1b), 100.5 (C-9), 104.2 (C-3), 115.5 (C-6), 115.7 (C-3a), 116.6 (C-4'), 118.9 (C-4a), 119.8 (C-1'), 120.5 (C-6'), 128.4 (C-4), 132.1 (C-5'), 143.5 (C-5), 150.6 (C-8a), 152.2 (C-9a), 153.4 (C-3'), 154.7 (C-2'), 156.9 (C-2), 160.4 (C-7), 189.3 (CHO). UV (EtOH) λ_max_ (lgε): 253 (3.81), 289 (2.52), 328 (3.11), 350 (3.08) nm. Anal. Calcd for C_30_H_30_N_2_O_6_: С, 70.02; Н, 5.88; N, 5.44; found С, 69.79; Н, 5.85; N, 5.16.

*(Z)-2-Isopropyl-3-(2,3,4-trimethoxystyryl)-2H-furo[3,2-g]chromene-3,7-dione* (**9**). Compound **9** (60 mg) was prepared from 2-isopropyl-3-[(2,3,4-trimethoxyphenyl)ethynyl]-7*H*-furo[3,2-*g*]chromene-7-one (**25**, 100 mg, 0.24 mmol) using the procedure described for **2**. Yield 60%, m.p. 94–97 °С (ether). IR (KBr, *ν*, cm^−^^1^): 3062, 3049, 2980, 2950, 1732, 1700, 1636, 1589, 1495, 1465, 1433, 1389, 1346, 1288, 1259, 1203, 1169, 1140, 1094, 1047, 1029, 1009, 962, 937, 903, 870, 831, 820, 781, 754, 700, 678, 650, 623, 602. ^1^H-NMR (600 MHz, CDCl_3_, δ_H_): 1.26 (d, *J* 6.9 Hz, 3Н, СН_3_), 1.28 [d, *J* = 6.9 Hz, 3Н, СН_3_), 3.16 [m, 1Н,СН-(СН_3_)_2_], 3.87 (s, 3Н, OСН_3_), 3.88 (s, 3Н, OСН_3_), 3.90 (s, 3Н, OСН_3_), 6.23 (d, *J* = 9.8 Hz, 1Н, Н-6), 6.40 (d, *J* = 8.8 Hz, 1Н, Н-1a), 6.71 (d, *J* = 8.8 Hz, 1Н, Н-1b), 6.83 (d, 1Н, *J =* 8.2 Hz, Н-5'), 6.90 (s, 1Н, Н-9), 7.19 (d, *J* = 8.2 Hz, 1Н, Н-6'), 7.77 (d, *J* = 9.8 Hz, 1Н, Н-5), 7.94 (s, 1Н, Н-4). ^13^C-NMR (150 MHz, CDCl_3_, δ_C_): 20.1 (CH_3_), 20.3 (CH_3_), 25.9 (CH), 55.3 (OCH_3_), 58.7 (OCH_3_), 60.6 (OCH_3_), 95.9 (C-3), 98.4 (C-9), 108.5 (C-3a), 114.0 (C-6), 115.5, 115.6 (C-5',4a), 123.0 (C-1'), 124.2 (C-6'), 125.9 (C-4), 130.0 (C-1a), 133.4 (C-1b), 141.5 (C-3'), 143.5 (C-5), 152.2 (C-8a), 153.0 (C-2'), 153.8 (C-4'), 154.7 (C-9a), 157.5 (C-2), 160.4 (C-7). UV (EtOH) λ_max_ (lgε): 240 (4.25), 252 (4.14), 296 (3.82), 352 (3.49) nm. Anal. Calcd for C_25_H_24_O_6_: С, 71.41; Н, 5.75; found С, 71.09; Н, 5.68. 

*(Z)-3-[(3,4-Dihydroxystyryl)-2-isopropyl-2H-furo[3,2-g]chromene-3,7-dione* (**10**). Compound **10** (42 mg) was prepared from 3-[(3,4-dihydroxyphenyl)ethynyl]-2-isopropyl-7*H*-furo[3,2-*g*]chromene-7-one (**26**, 100 mg, 0.27 mmol) using the procedure described for **3**. Yield 42%, a yellow oil. IR (KBr, *ν*, cm^−^^1^): 3450, 3050, 2980, 1732, 1717, 1670, 1610, 1603, 1514, 1470, 1431, 1412, 1364, 1279, 1254, 1192, 1140, 1110, 1086, 1041, 990, 975, 928, 910, 860, 849, 820, 770, 754, 740, 648, 627, 601. ^1^H-NMR (400 MHz, CDCl_3_, δ_H_): 1.36 (d, *J* = 6.9 Hz, 3Н, (СН_3_), 1.37 (d, *J* = 6.9 Hz, 3Н, (СН_3_), 3.25 [m, 1Н, СН-(СН_3_)_2_], 3.78 (br.s, 2Н, OH), 6.30 (d, *J* = 9.8 Hz, 1Н, Н-6), 6.39 (d, *J* = 9.0 Hz, 1Н, Н-1a), 6.65 (d, *J* = 9.0 Hz, 1Н, Н-1b), 6.80 (d, *J* = 8.0 Hz, 1Н, Н-5'), 6.89 (s, 1Н, Н-9), 7.36 (d, *J* = 1.6 Hz, 1Н, Н-2'), 7.42 (dd, *J* = 8.0 and 1.6 Hz, 1Н, Н-6'), 7.79 (d, *J* = 9.8 Hz, 1Н, Н-5), 7.80 (s, 1Н, Н-4). ^13^C-NMR (100 MHz, CDCl_3_, δ_C_): 20.1 (CH_3_), 20.3 (CH_3_), 24.4 (CH), 96.0 (C-3), 98.4 (C-9), 106.6 (C-3a), 114.0 (C-6), 114.9, 115.1 (C-1',5'), 115.5 (C-2'), 117.2 (C-4a), 117.6 (C-6'), 125.6 (C-4), 130.9 (C-1a), 133.8 (C-1b), 143.5 (C-5), 146.5 (C-3'), 147.9 (C-4'), 152.2 (C-8a), 153.0 (C-9a), 156.4 (C-2), 160.3 (C-7). UV (EtOH) λ_max_ (lgε): 252 (4.47), 287 (4.02), 350 (3.62) nm. Anal. Calcd for C_22_H_18_O_5_: С, 72.92; Н, 5.01; found С, 72.78; Н, 4.88. 

*(Z)-3-(5-Formyl-3-hydroxy-3-methoxystyryl)-3-isopropyl-2H-furo[3,2-g]chromene-3,7-dione* (**11**)*.* Compound **11** (61 mg) was prepared from 3-[(5-formyl-3-hydroxy-3-methoxyphenyl)ethynyl]-2-isopropyl-7*H*-furo[3,2-*g*]chromene-7-one (**27**, 100 mg, 0.24 mmol) using the procedure described for **3**. Yield 60%, m.p. 104–105 °С (ether). IR (KBr, *ν*, cm^−^^1^): 3435, 3063, 3042, 2960, 2925, 2853, 2808, 1739, 1726, 1714, 1629, 1600, 1576, 1510, 1500, 1464, 1440, 1394, 1355, 1334, 1300, 1250, 1230, 1176, 1157, 1144, 1105, 1032, 978, 935, 915, 893, 831, 814, 761, 744, 720, 668.^ 1^H-NMR (400 MHz, CDCl_3_, δ_H_): 1.43 (d, *J =* 6.9 Hz, 3Н, СН_3_), 1.46 (d, *J* = 6.9 Hz, 3Н, СН_3_), 3.34 [m, 1Н, СН-(СН_3_)_2_], 3.98 (s, 3Н, OСН_3_), 6.39 (d, *J* = 9.8 Hz, 1Н, Н-6), 6.41 (d, *J* = 1.8 Hz, 1Н, Н-6'), 6.48 (d, *J* = 8.8 Hz, 1Н, Н-1a), 6.82 (d, *J* = 8.8 Hz, 1Н, Н-1b), 7.05 (s, 1Н, Н-9), 7.10 (br.s, 1Н, OН), 7.59 (d, *J* = 1.8 Hz, 1Н, Н-4'), 7.64 (s, 1Н, Н-4), 7.86 (d, *J* = 9.8 Hz, 1Н, Н-5), 10.03 (1Н, s, CНO). ^13^C-NMR (100 MHz, CDCl_3_, δ): 20.1 (CH_3_), 20.3 (CH_3_), 25.8 (CH), 55.9 (OCH_3_), 99.1 (C-3), 104.1 (C-9), 115.4 (C-3a), 115.9 (C-6), 116.5 (C-4'), 117.1 (C-4a), 118.6 (C-1'), 121.4 (C-6'), 126.8 (C-4), 128.3 (C-1b), 132.1 (C-1a), 132.6 (C-5'), 143.4 (C-5), 149.9 (C-3'), 152.1 (C-8a), 152.9 (C-9a), 154.4 (C-2'), 156.8 (C-2), 160.3 (C-7), 188.2 (СНО). UV (EtOH) λ_max_ (lgε): 252 (4.26), 284 (3.65), 346 (3.53) nm. Anal. Calcd for C_24_H_20_O_6_: С, 70.92; Н, 4.88; found С, 71.28; Н, 4.98.

*(Z)-3-[(3,4-Dihydroxystyryl)-2-isopropyl-9-[(4-methylpiperazin-1-yl)methyl]-2H-furo[3,2-g]-chrom-ene-3,7-dione* (**12**). The compound **12** (49 mg) was prepared from 3-[(3,4-dihydroxyphenyl)ethynyl]-2-isopropyl-9-[(4-methylpiperazin-1-yl)methyl]-7*H*-furo[3,2-*g*]-chromene-7-one (**28**, 123 mg, 0.26 mmol) using the procedure described for **3**. Yield 40%, a yellow oil. IR (KBr, *ν*, cm^−^^1^): 3433, 3060, 3049, 2955, 2922, 2851, 1732, 1660, 1628, 1580, 1496, 1467, 1431, 1389, 1349, 1310, 1286, 1250, 1213, 1198, 1140, 1115, 1071, 1047, 988, 957, 905, 870, 820, 790, 768, 752, 736, 725, 708, 690, 675, 650.^ 1^H-NMR (600 MHz, CDCl_3_, δ_H_): 1.35 (d, *J*= 7.0 Hz, 3Н, СН_3_), 1.37 (d, *J* = 7.0 Hz, 3Н, СН_3_), 2.28 (s, 3Н, СН_3_), 2.48 (m, 4H, H-3'',5''), 2.89 (m, 4H, H-2'',6''), 3.25 [m, 1Н, СН-(СН_3_)_2_], 4.57 (d, *J* = 9.8 Hz, 1H, CH_2_), 4.62 (d, *J* = 9.8 Hz, 1H, CH_2_), 6.28 (d, *J* = 9.7 Hz, 1Н, Н-6), 6.37 (d, *J* = 9.0 Hz, 1Н, Н-1a), 6.77 (d, *J* = 8.3 Hz, 1Н, Н-5'), 6.95 (d, *J* = 9.0 Hz, 1Н, Н-1b), 7.38 (d, *J* = 1.8 Hz, 1Н, Н-2'), 7.49 (dd, *J* = 8.0 and 1.8 Hz, 1Н, Н-6'), 7.65 (d, *J* = 8.0 Hz, 1Н, Н-5'), 7.78 (s, 1Н, Н-4), 7.91 (d, 1Н, *J* = 9.7 Hz, Н-5), 8.17 (br.s, 2Н, OH). ^13^C-NMR (100 MHz, CDCl_3_, δ_C_): 19.9 (CH_3_), 20.2 (CH_3_), 25.6 (CH), 42.4 (СН_3_), 48.2 (СН_2_), 52.8 (C-3'',5''), 51.3 (C-2'',6''), 98.9 (C-3), 103.9 (C-9), 115.2 (C-3a), 116.3 (C-6), 117.0 (C-5'), 117.4 (C-4a), 120.0 (C-1'), 126.1 (C-2'), 127.8 (C-6'), 128.1 (C-4), 130.6, 131.6 (C-1a,1b), 143.2 (C-5), 147.1 (C-3'), 148.1 (C-4'), 151.9 (C-9a), 152.7 (C-8a), 157.7 (C-2), 160.0 (C-7). UV (EtOH) λ_max_ (lgε): 252 (4.42), 275(sh), 288 (3.91), 306 (3.84), 322 (sh), 354 (3.28) nm. Anal. Calcd for C_28_H_30_N_2_O_5_: С, 70.87; Н, 6.37; N, 5.90; found С, 71.02; Н, 6.33; N, 5.81.

*(Z)-2-(5-Formyl-3-hydroxy-3-methoxystyryl)-3-isopropyl-9-[(4-methylpiperazin-1-yl)methyl]-2H-furo-[3,2-g]chromene-3,7-dione* (**13**). Compound **13** (56 mg) was prepared from 3-[(5-formyl-3-hydroxy-3-methoxyphenyl)ethynyl]-2-isopropyl-9-[(4-methylpiperazin-1-yl)methyl]-7*H*-furo[3,2-*g*]chromen-7-one (**29**, 133 mg, 0.26 mmol) using the procedure described for **3**. Yield 42%, m.p. 100–102 °С (ether). IR (KBr, *ν*, cm^−^^1^): 3402, 3200, 3061, 3049, 2968, 2954, 2922, 2874, 2818, 1732, 1705, 1628, 1580, 1510, 1497, 1466, 1431, 1410, 1389, 1348, 1300, 1287, 1250, 1214, 1198, 1140, 1115, 1070, 1047, 1020, 975, 956, 910, 870, 820, 780, 752, 737, 720, 700, 660.^ 1^H-NMR (400 MHz, CDCl_3_, δ_H_): 1.34 (d, *J* = 6.9 Hz, 3Н, (СН_3_), 1.39 (d, *J* = 6.9 Hz, 3Н, (СН_3_), 2.28 (s, 3Н, СН_3_), 2.41 (m, 4H, H-3'',5''), 2.65 (m, 4H, H-2'',6''), 3.24 [m, 1Н, СН-(СН_3_)_2_], 4.03 (s, 3Н, OСН_3_), 4.48 (d, *J* = 9.8 Hz, 1H, CH_2_), 4.52 (d, *J* = 9.8 Hz, 1H, CH_2_), 6.30 (d, *J* = 9.8 Hz, 1Н, Н-6), 6.38 (d, *J* = 9.1 Hz, 1Н, Н-1a), 6.45 (d, *J* = 1.8 Hz, 1Н, Н-6'), 6.95 (d, *J* = 9.1 Hz, 1Н, Н-1b), 7.48 (d, *J* = 1.8 Hz, 1Н, Н-4'), 7.68 (s, 1Н, Н-4), 7.77 (d, *J* = 9.8 Hz, 1Н, Н-5), 9.95 (br.s, 1Н, CНO). ^13^C-NMR (100 MHz, CDCl_3_, δ_C_): 19.9 (CH_3_), 20.1 (CH_3_), 25.8 (CH), 42.5 (СН_3_), 48.3 (СН_2_), 51.4 (C-2'',6''), 52.8 (C-3'',5''), 58.3 (OCH_3_), 102.6 (C-3), 104.0 (C-9), 115.4 (C-3a), 115.8 (C-6), 116.4 (C-4a), 118.8 (C-4'), 119.9 (C-1'), 120.0 (C-6'),125.2 (C-4), 128.6 (C-1a),131.9 (C-1b), 133.1 (C-5'), 145.1 (C-5), 145.6 (C-3'), 151.3 (C-8a), 152.0 (C-9a), 154.6 (C-2'), 156.8 (C-2), 158.9 (C-7), 189.2 (СНО). UV (EtOH) λ_max_ (lgε): 252 (4.59), 290 (3.82), 336 (sh), 353(3.42) nm. Anal. Calcd for C_30_H_32_N_2_O_6_: С, 69.75; Н, 6.24; N, 5.42; found С, 69.52; Н, 6.38; N, 5.35.

*2-Isopropyl-3-[(trimethylsilyl)ethynyl]-7Н-furo[3,2-g]chromen-7-оne* (**31**). To a solution of oreoselone triflate **23** (200 mg, 0.5 mmol) and (trimethylsilyl)acetylene (**30**, 73 mg, 0.75 mmol) in benzene (5 mL) was added CuI (1.5 mg, 2 mol %), Pd(PPh_3_)_2_Cl_2_ (11 mg, 4 mol %), and Et_3_N (0.076 mL, 0.55 mmol; 1.1 equiv) under argon. The reaction mixture was stirred at 80 °C for 10 h (TLC). The mixture was cooled, and 5 mL of water was added. The separated water layer was extracted with methylene chloride (5 × 4 mL). The combined organic extracts was washed with water, dried over MgSO_4_, filtered, and concentrated under reduced pressure. The residue was subjected to column chromatography on silica gel. Eluting with chloroform and crystallization from diethyl ether gave 90 mg (58%) of compound **31**, m.p. 94–96 °С. IR (KBr, *ν*, cm^−^^1^): 3435, 2962, 2925, 2152, 1730, 1625, 1597, 1577, 1485, 1431, 1388, 1355, 1284, 1249, 1211, 1197, 1139, 1101, 1068, 1047, 914, 869, 754, 688. ^1^H-NMR (600 MHz, CDCl_3_, δ_H_): 0.16 [9Н, s, (СН3)3Si], 1.35 (d, *J =* 7 Hz, 3Н, СН_3_), 1.38 (d, *J =* 7 Hz, 3Н, СН_3_), 3.25 [1H, m, СH(СН3)2], 6.40 (d, *J* = 9.6 Hz, 1Н, Н-6), 7.40 (s, 1Н, Н-9), 7.57 (s, 1Н, Н-4), 7.79 (d, *J* = 9.6 Hz, 1Н, Н-5). ^13^C-NMR (150 MHz, CDCl_3_, δ_C_): 9.5 (3×СН_3_), 20.1 (СН_3_), 20.3 (СН_3_), 27.3 (СН), 89.2 (С-1a), 94.8 (С-1b), 96.2 (С-3), 98.6 (С-9), 106.9 (С-3а), 114.3 (С-6), 115.7 (С-4а), 115.8 (С-4), 116.8 (С-3), 142.7 (С-5), 152.4 (С-8а), 153.2 (С-9a), 159.0 (С-2), 160.8 (С-7). UV (EtOH) λ_max_ (lgε): 222 (4.36), 251 (4.07), 294 (2.74), 338 (2.64) nm. Anal. Calcd for C_19_H_20_O_3_Si: С, 70.34; Н, 6.21; Si 8.66; found С, 69.98; Н, 5.99; Si, 8.35.

*3-Ethynyl-2-isopropyl-7H-furo[3,2-g]chromen-7-one* (**32**). To a solution of compound **31** (100 mg, 0.3 mmol) in methanol (5 mL) were added CsF (230 mg, 1.5 mmol) and benzyltrimethylammonium chloride (28 mg, 0.15 mmol). The mixture was stirred at rt for 10 h in under argon (TLC). Then 10 mL of water was added and the mixture was extracted with methylene chloride (5 × 4 mL). The combined extract was washed with water, dried over MgSO_4_, filtered, and concentrated under reduced pressure. The residue was subjected to column chromatography on silica gel. Eluting with chloroform and crystallization from diethyl ether gave compound **32** (50 mg, 66%). M.p. 82–83 °С (ether). IR (KBr, *ν*, cm^−^^1^): 3435, 3059, 2979, 2935, 2877, 2185, 1732, 1685, 1625, 1577, 1471, 1433, 1388, 1321, 1286, 1249, 1211, 1197, 1137, 1116, 1047, 869, 819, 721, 694. ^1^H-NMR (400 MHz, CDCl_3_, δ_H_): 1.25 (d, *J* *=* 7 Hz, 3Н, СН_3_), 1.29 (d,3Н, *J* = 7 Hz, СН_3_), 2.37 (s, 1H, ≡CH), 3.19 [m, 1H, СH(СН_3_)_2_], 6.32 (d, *J* = 9.6 Hz, 1Н, Н-6), 7.33 (s, 1Н, Н-9), 7.50 (s, 1Н, Н-4), 7.71 (d, *J* = 9.6 Hz, 1Н, Н-5). ^13^C-NMR (100 MHz, CDCl_3_, δ_C_): 20.2 (СН_3_), 20.4 (СН_3_), 25.8 (СН), 80.5 (С-1a), 87.6 (С-1b), 96.1 (С-3), 100.4 (С-9), 107.3 (С-3а), 115.6 (С-6), 116.0 (С-4а), 116.4 (С-4), 116.6 (С-3), 143.5 (С-5), 152.2 (С-8а), 153.1 (С-9a), 156.9 (С-2), 160.2 (С-7). UV (EtOH) λ_max_ (lgε): 224 (3.91), 250 (3.94), 285 (sh), 335 (3.34) nm. Anal. Calcd for C_16_H_12_O_3_: С, 76.18; Н, 4.79; found С, 76.31; Н, 5.09.

### 3.3. Cell Culture and Cytotoxicity Assay

The human cancer cells of the MT-4, CEM-13 (the cells of T-cellular human leucosis), and U-937 (human monocytes) were used in this study. The cells were cultured in the RPMI-1640 medium that contained 10% embryonic calf serum, L-glutamine (2 mmol/L), gentamicin (80 lg/mL), and lincomycin (30 mg/mL) in a CO_2_ incubator at 37 °C. The tested compounds were dissolved in DMSO and added to the cellular culture at the required concentrations. Three wells were used for each concentration. The cells which were incubated without the compounds were used as a control. Cells were placed on 96-well microliter plates and cultivated at 37 °С C in 5% CO_2_/95% air for 72 h. The cell viability was assessed through an MTT [3-(4,5-dimethylthiazol-2-yl)-2,5-phenyl-2H-tetrazolium bromide] conversion assay. 1% MTT was added to each well. Four hours later DMSO was added and mixed for 15 min. Optical density (*D*) of the samples was measured on a BioRad 680 spectrophotometer Microplate Reader (BioRad, Hercules, CA, USA) at the wavelength of 570 nm. The 50% cytotoxic dose (CTD50) of each compound (*i.e.*, the compound concentration that causes the death of 50% of cells in a culture, or decreases the optical density twice as compared to the control wells) was calculated from the data obtained. Statistical processing of the results was performed using the Microsoft Excel-2007, STATISTICA 6.0, and GraphPad Prism 5.0 programs. The results are given as an average value ± a deviation from the average. Reliability of differences (*p*) was estimated using the Student t test. The differences with *p* < 0.05 were considered as reliable. The experimental results are given as the data average values obtained from three independently conducted experiments.

## 4. Conclusions

A series of original furocoumarin derivatives having 2-(*Z*)- or 3-(*Z*)-styryl substitution in their structures have been synthesized. The cytotoxic activity of the resulting compounds against several cancer lines have been determined in the conventional MTT assay. The cytotoxicity data of compounds **2**–**13** demonstrate that they exhibit anticancer activity in micromolar range. Structure-activity comparison provides evidence that а 2-(*Z*)-styryl substitution in the furocoumarin scaffold is preferred for cytotoxicity over the subsequent 3-(*Z*)-styryl substitution; the (4-methylpiperazin-1-ylmethyl) substitution in the 9-position of 3-styrylfurocoumarins increases the cytotoxic activity in MT-4 and U-937 cell lines. The biological results for the furocoumarin analogs of CA-4 **1**, reported herein, shown that the structural modification of furocoumarins with the introduction of (*Z*)-styryl moiety may prove of great importance to obtain cytotoxic anti-cancer agents.
